# Supplementary material for: Resolving active species during the carbon monoxide oxidation over Pt(111) on the microsecond timescale
Source: Nat Commun. 2025 Jan 31;16:1216. doi: 10.1038/s41467-025-56576-5 (PMC11785939; doi:10.1038/s41467-025-56576-5)
Supplement: Supplementary file 1 — Supplementary Information [file 41467_2025_56576_MOESM1_ESM.pdf]

SUPPLEMENTARY INFORMATION

Resolving Active Species during the Carbon Monoxide Oxidation over Pt(111) on the Microsecond Timescale

Calley N. Eads[1], Weijia Wang[1], Ulrike Küst[2,3], Julia Prumbs[2], Robert H. Temperton[1], Mattia Scardamaglia[1], Joachim Schnadt[1,2,3], Jan Knudsen[1,2,3], Andrey Shavorskiy[1]*

[1]MAX IV Laboratory, Lund University, Lund, Sweden
[2]Division of Synchrotron Radiation Research, Department of Physics, Lund University, Lund, Sweden
[3]NanoLund, Lund University, Lund, Sweden

*email: andrey.shavorskiy@maxiv.lu.se

Supplementary Table 1. A non-exhaustive list of specific time resolutions in leading time-resolved techniques coupled to spectroscopic or scattering method/s in numerous publications. TAP, temporal analysis of products; SSITKA, steady-state isotopic transient kinetic analysis; MBS, molecular beam scattering; MES, modulation excitation spectroscopy; APXPS, ambient pressure X-ray photoelectron spectroscopy; IR, infrared spectroscopy; DRIFTS, diffuse reflectance infrared Fourier transform spectroscopy; IRRAS, infrared reflection-absorption spectroscopy; NAP-VMI, near ambient pressure velocity map imaging; XAS, X-ray absorption spectroscopy; EDXAS, energy dispersive XAS; XRD, X-ray diffraction; FTIR, Fourier transform IR.

TR-technique	Coupled spectroscopic or scattering method	Time resolution	Ref
TAP	APXPS	70 s	1
	APXPS	300 ms	2
SSITKA	IR	120 s	3
	FTIR	60 s	4
	DRIFTS	50 s	5
	DRIFTS	15 s	6
	DRIFTS	10 s	7
MB	IRRAS	~1.5 s	8
	IRRAS	240 ms	9
	XPS	500 μs	10
	NAP-VMI	10 μs	11,12
MES	DRIFTS	300 s	13
	XAS	4 s	14
	DRIFTS	1.9 s	15
	APXPS	1 s	16
	XRD	500 ms	17
	XRD	500 ms	18
	DRIFTS	470 ms	18
	DRIFTS	433 ms	19
	FTIR	400 ms	20
	DRIFTS	333 ms	17
	DRIFTS	300 ms	21
	EDXAS	260 ms	22
	XAS	260 ms	21
	XAS	250 ms	19
	XAS	250 ms	23
	DRIFTS	242 ms	24
	EDXAS	≥190 ms	25
	EDXAS	100 ms	26
	DRIFTS	≥20 ms	27
tr-APXPS		60 ms	28
		40 μs	this work
		20 μs	29

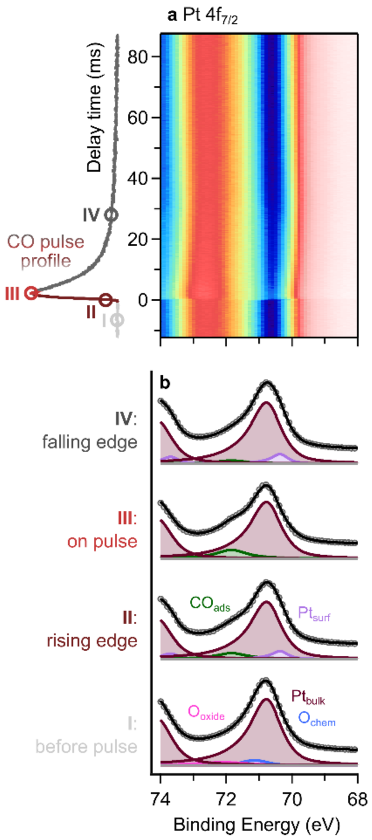

Supplementary Fig. 1. Time-resolved APXPS of CO oxidation of Pt(111) with 40 μs time resolution when pulsing CO gas at 10 Hz into a stream of O_2 while monitoring a *Pt 4f* with b representative fits displaying original fits that include Pt_{bulk} contribution.

Supplementary Table 2. Binding energies of all fitted components from tr-APXP spectra in Fig. 2f-j with their associated color and reference to relevant findings. Gas phase (GP) binding energies are recorded from the before pulse region and shift by 0.1-0.2 eV with the CO pulse suggesting a change in surface composition on the CO pulse and reflecting the sensitivity of gas phase species to work function changes[30]. *See Supplementary Fig. 4 for Pt_{surf} binding energy trends that shift from 70.34-70.26 eV.

Core-level	Assignment	Color	Binding Energy (eV)		Refs
C 1s GP	CO_2(g)	Gold	291.6		31,32
	CO(g)	Red	290.2		31–33
O 1s GP	O_2(g)	purple & light blue	538.4<br>537.3		31,32,34
	CO(g)	Red	536.5		31,32,34
	CO_2(g)	Gold	535.1		31,32,34
O 1s	CO_{top}	Green	532.5		31–36
	O_{oxide}	Pink	531.6		31,32,37–39
	CO_{bridge}	Peach	531.1		31–36
	O_{chem}	Blue	529.7		31,32,34,36,39–41
C 1s	CO_{top}	Green	286.7		31–33,36
	CO_{bridge}	Peach	285.9		31–33,36
Pt 4f$_{7/2}$	O_{oxide}	Pink	72.1	Pt_{bulk} +1.4	37,39,42–44
	CO_{ads}	dark green	71.8	Pt_{bulk} +1.1	32,33,35,36
	O_{chem}	Blue	71.1	Pt_{bulk} +0.4	36,40,42
	Pt_{bulk}	Brown	70.7		40
	Pt_{surf}	Lavender	70.3*	Pt_{bulk} -0.4	32,33,36,39–41

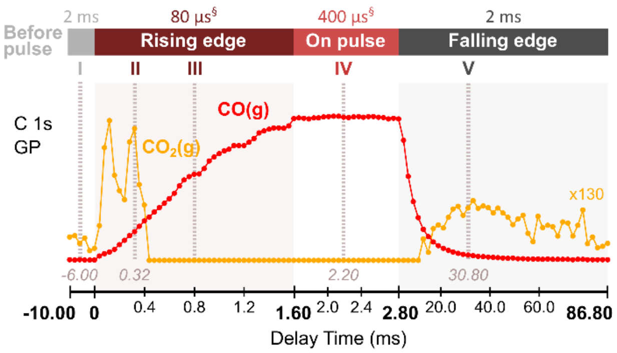

Supplementary Fig. 2. Areas trend data of *C 1s* GP in the CO oxidation of Pt(111) when pulsing CO at 10 Hz into a stream of O_2 gas. CO(g) and CO_2(g) show similar behavior to trends separately assessed in *O 1s* GP spectra presented in Fig. 2a of the main text.

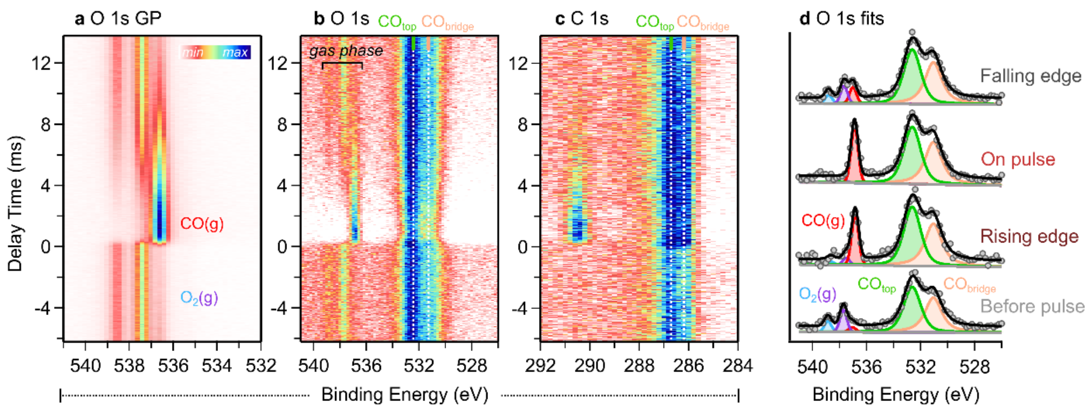

Supplementary Fig. 3. tr-APXPS of CO oxidation of Pt(111) when pulsing CO at 50 Hz into a stream of O_2 where CO_2 production was purposefully switched off by increasing the CO valve opening time leading to deactivation of the catalyst. 2D plots of a *O 1s* GP, b *O 1s*, and c *C 1s* demonstrate a CO poisoning effect further evidenced in d *O 1s* fits where only CO_{ads} were observed.

Supplementary Note 1. Comparison of steady-state and modulating conditions

A typical experiment to assess the function of a catalyst involves flowing gases into a reactor and monitoring reaction products via mass spectrometry. Here, we compare conventional steady-state experiments with our pulsing approach to study how transient changes impact CO_2 production in C 1s gas phase spectra at various O_2:CO ratios (Supplementary Fig. 4). By varying the way CO gas is dosed, we examined ratios from 7.5:1 to 60:1 O_2:CO and observed notable differences between pulsing CO at 10 Hz (Supplementary Fig. 4a) and a continuous CO flow (Supplementary Fig. 4b). All spectra are time averaged and taken in time-resolved mode to ensure consistency. Upon analyzing *C 1s* GP spectra and extracting CO_2 intensities (Supplementary Fig. 4c) and normalized CO_2 intensities (Supplementary Fig. 4d), CO_2 production remains relatively steady during pulsing, with a slight increase in more CO-rich environments. Under constant flow, CO_2 production peaks at 30:1 O_2:CO ratio. Remarkably, at 7.5:1, CO_2 production is highest during CO pulsing, whereas CO_2 formation ceases entirely under constant flow. To explain the Pt catalyst's behavior at this ratio, we analyzed surface species signals in the *C 1s*, *O 1s*, and *Pt 4f$_{7/2}$* core levels (Supplementary Fig. 4e). In constant flow, CO adsorbates dominate, poisoning the Pt catalyst and preventing O_2 molecules from interacting and dissociating, thus halting CO oxidation. In contrast, pulsing allowed CO_{ads} and O

adsorbates to coexist, maintaining a balanced interaction that promoted CO_2 production. These experiments highlight another method to control catalytic activity simply by pulsing or flowing CO.

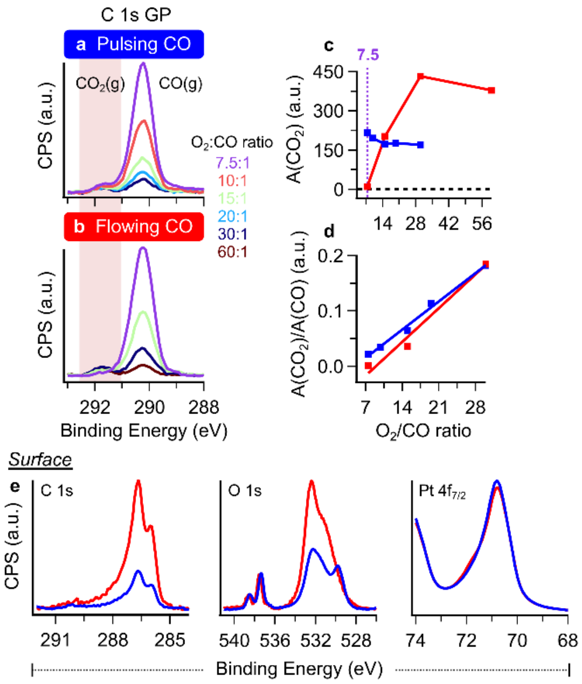

Supplementary Fig. 4. Activity changes when pulsing vs. flowing CO gas into stream of O_2. Assessing the effect of **a** pulsing CO at 10 Hz vs. b flowing CO into a stream of O_2 gas varying O_2:CO ratios while monitoring CO_2 production in time-averaged *C 1s* gas phase spectra. c Comparing CO_2 areas (normalized in d) from pulsed (blue) and flow (red) experiments reveals near constant reactivity under pulsed conditions and notably shows CO_2 production at 7.5:1 O_2:CO ratio when CO_2 is not detected in flow experiments. e At this ratio of 7.5:1 O_2:CO, time-averaged XP surface spectra of *C 1s*, *O 1s*, and *Pt 4f*$_{7/2}$ show significant differences in surface species involved in the reaction, i.e. CO-rich under flow conditions and a mixture of CO and O species under pulsed conditions.

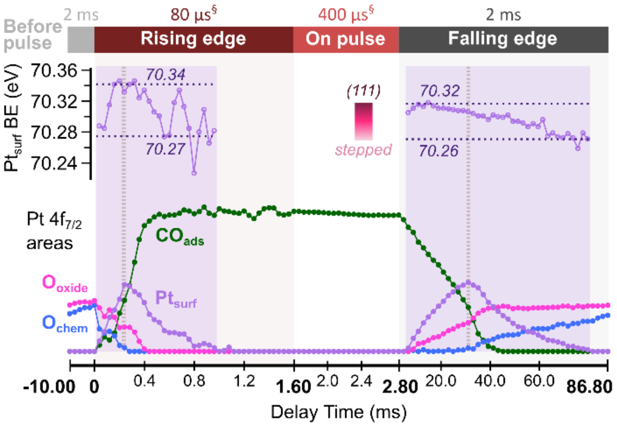

Supplementary Fig. 5**.** Pt_{surf} binding energy trend (top) with respect to *Pt 4f*$_{7/2}$ areas trend (bottom) over time. The binding energy of Pt_{surf} is sensitive to changes in the Pt surface. Terraced surfaces like Pt(111) will typically have a binding energy change of 0.4 eV with respect to Pt_{bulk} whereas stepped Pt surfaces are greater, such as, 0.50 eV for Pt(997)[45] and 0.65 eV for Pt(557)[46]. We observe a shift from 0.41-0.43 eV to 0.48-0.49 eV suggesting a change in the Pt surface from (111)-type surface to a more step-like surface, respectively. This observation supports a change in the CO oxidation mechanism from CO_{ads} reacting with O_{chem} at the start followed by less reactive, raised O_{oxide}.

Supplementary Table 3. Fitting parameters used in 10 Hz and 50 Hz datasets.

Core-level		Feature	10 Hz BE (eV)	10 Hz ΔBE (eV)	10 Hz FWHM	10 Hz GL	50 Hz BE (eV)	50 Hz FWHM	50 Hz GL
C 1s	surf	CO_{bridge}*	285.9		0.49	0.59	285.9	0.49	0.59
		CO_{top}*	286.6		0.61	0.76	286.6	0.61	0.76
	GP	CO(g)	290.2		0.80	0.59	-	-	-
		CO_2(g)	291.6		0.49	0	-	-	-
O 1s	surf	O_{chem}	529.7		1.19	0.19	-	-	-
		CO_{bridge}	531.1		1.51	0.83	531.1	1.49	0.83
		O_{oxide}	531.6		2.09	0.28	-	-	-
		CO_{top}	532.5		1.38	0.49	532.6	1.50	0.49
	GP	CO_2(g)	535.2		0.60	0	-	-	-
		CO(g)	536.4		0.49	0.82	536.6	0.49	0.82
		O_2(g)	537.2		0.51	0.82	537.4	0.51	0.82
			538.4		0.51	0.82	538.6	0.51	0.82
Pt 4f$_{7/2}$	surf	Pt_{surf}**	70.3	Pt_{bulk}-0.4	0.54	0.73	-	-	-
		Pt_{bulk}**	70.7		1.02	0.73	-	-	-
		O_{chem}	71.1	Pt_{bulk}+0.4	0.90	0.30	-	-	-
		CO_{ads}	71.8	Pt_{bulk}+1.1	1.13	1	-	-	-
		O_{oxide}	72.1	Pt_{bulk}+1.4	1.83	0.30	-	-	-

*Asymmetric contributions: asym1 = 0.29, asym2 = 0.82 used in EccentricXPS package in IgorPro.
**Asymmetric contributions: asym1 = 0.23, asym2 = 0.42.

Supplementary References

1. Redekop, E. A. et al. Aligning time-resolved kinetics (TAP) and surface spectroscopy (AP-XPS) for a more comprehensive understanding of ALD-derived 2D and 3D model catalysts. Faraday Discuss. 236, 485–509 (2022).

2. Redekop, E. A. et al. Synchronizing gas injections and time-resolved data acquisition for perturbation-enhanced APXPS experiments. Rev. Sci. Instrum. 92, 44101 (2021).

3. El-Roz, M., Bazin, P., Daturi, M. & Thibault-Starzyk, F. Operando infrared (IR) coupled to steady-state isotopic transient kinetic analysis (SSITKA) for photocatalysis: Reactivity and mechanistic studies. ACS Catal. 3, 2790–2798 (2013).

4. Bazin, P., Thomas, S., Marie, O. & Daturi, M. New insights into the methanol oxidation mechanism over Au/CeO2 catalyst through complementary kinetic and FTIR operando SSITKA approaches. Catal. Today 182, 3–11 (2012).

5. Kalamaras, C. M., Olympiou, G. G. & Efstathiou, A. M. The water-gas shift reaction on Pt/γ-Al2O3 catalyst: Operando SSITKA-DRIFTS-mass spectroscopy studies. Catal. Today 138, 228–234 (2008).

6. Rezvani, A. et al. CO2 Reduction to Methanol on Au/CeO2 Catalysts: Mechanistic Insights from Activation/Deactivation and SSITKA Measurements. ACS Catal. 10, 3580–3594 (2020).

7. Goguet, A., Meunier, F. C., Tibiletti, D., Breen, J. P. & Burch, R. Spectrokinetic investigation of reverse water-gas-shift reaction intermediates over a Pt/CeO2 catalyst. J. Phys. Chem. B 108, 20240–20246 (2004).

8. Johánek, V., Schauermann, S., Laurin, M., Libuda, J. & Freund, H. J. Site Occupation and Activity of Catalyst Nanoparticles Monitored by In Situ Vibrational Spectroscopy. Angew. Chemie Int. Ed. 42, 3035–3038 (2003).

9. Hoffmann, J., Schauermann, S., Johánek, V., Hartmann, J. & Libuda, J. The kinetics of methanol oxidation on a supported Pd model catalyst: molecular beam and TR-IRAS experiments. J. Catal. 213, 176–190 (2003).

10. Höfert, O., Gleichweit, C., Steinrück, H. P. & Papp, C. Ultrafast x-ray photoelectron spectroscopy in the microsecond time domain. Rev. Sci. Instrum. 84, 93103 (2013).

11. Chien, T. E., Hohmann, L. & Harding, D. J. Near-ambient pressure velocity map imaging. J. Chem. Phys. 157, 34201 (2022).

12. Chien, T. E., Hohmann, L. & Harding, D. J. Time-resolved surface reaction kinetics in the pressure gap. Faraday Discuss. (2024) doi:10.1039/D3FD00158J.

13. Gau, A., Hack, J., Maeda, N. & Meier, D. M. Operando Spectroscopic Monitoring of Active Species in CO2 Hydrogenation at Elevated Pressure and Temperature: Steady-State versus Transient Analysis. Energy and Fuels 35, 15243–15246 (2021).

14. Vogt, C. et al. Unravelling structure sensitivity in CO2 hydrogenation over nickel. Nat. Catal. 2017 12 1, 127–134 (2018).

15. Maeda, N., Meemken, F., Hungerbühler, K. & Baiker, A. Spectroscopic Detection of Active Species on Catalytic Surfaces: Steady-State versus Transient Method. Chimia (Aarau). 66, 664 (2012).

16. Roger, M. et al. Improving time-resolution and sensitivity of in situ X-ray photoelectron spectroscopy of a powder catalyst by modulated excitation. Chem. Sci. 14, 7482–7491 (2023).

17. Newton, M. A., Michiel, M. Di, Kubacka, A. & Fernández-García, M. Combining time-resolved hard X-ray diffraction and diffuse reflectance infrared spectroscopy to illuminate CO dissociation and transient carbon storage by supported Pd nanoparticles during CO/NO cycling. J. Am. Chem. Soc. 132, 4540–4541 (2010).

18. Ferri, D. et al. Revealing the Dynamic Structure of Complex Solid Catalysts Using Modulated Excitation X-ray Diffraction. Angew. Chemie Int. Ed. 53, 8890–8894 (2014).

19. Newton, M. A., Ferri, D., Smolentsev, G., Marchionni, V. & Nachtegaal, M. Kinetic Studies of the Pt Carbonate-Mediated, Room-Temperature Oxidation of Carbon Monoxide by Oxygen over Pt/Al2O3 Using Combined, Time-Resolved XAFS, DRIFTS, and Mass Spectrometry. J. Am. Chem. Soc. 138, 13930–13940 (2016).

20. Kritzenberger, J. & Wokaun, A. Time resolved FTIR study of the catalytic CO oxidation under periodic variation of the reactant concentration. J. Mol. Catal. A Chem. 118, 235–245 (1997).

21. Editor, G. et al. First steps in combining modulation excitation spectroscopy with synchronous dispersive EXAFS/DRIFTS/mass spectrometry for in situ time resolved study of heterogeneous catalysts. Phys. Chem. Chem. Phys. 12, 5634–5646 (2010).

22. Ferri, D., Newton, M. A. & Nachtegaal, M. Modulation excitation X-ray absorption spectroscopy to probe surface species on heterogeneous catalysts. Top. Catal. 54, 1070–1078 (2011).

23. Gaur, A. et al. Using Transient XAS to Detect Minute Levels of Reversible S-O Exchange at the Active Sites of MoS2-Based Hydrotreating Catalysts: Effect of Metal Loading, Promotion, Temperature, and Oxygenate Reactant. ACS Catal. 12, 633–647 (2022).

24. Aguirre, A. & Collins, S. E. Selective detection of reaction intermediates using concentration-modulation excitation DRIFT spectroscopy. Catal. Today 205, 34–40 (2013).

25. Nilsson, J. et al. Chemistry of Supported Palladium Nanoparticles during Methane Oxidation. ACS Catal. 5, 2481–2489 (2015).

26. Becker, E., Carlsson, P. A., Kylhammar, L., Newton, M. A. & Skoglundh, M. In situ spectroscopic investigation of low-temperature oxidation of methane over alumina-supported platinum during periodic operation. J. Phys. Chem. C 115, 944–951 (2011).

27. Wang, X. & Bürgi, T. Influence of the Time Scale on the Reaction Mechanism of CO Oxidation over a Au/TiO2 Catalyst. Angew. Chemie Int. Ed. 62, e202300146 (2023).

28. Knudsen, J. et al. Stroboscopic operando spectroscopy of the dynamics in heterogeneous catalysis by event-averaging. Nat. Commun. 12, 1–8 (2021).

29. Shavorskiy, A. et al. Gas Pulse-X-Ray Probe Ambient Pressure Photoelectron Spectroscopy with Submillisecond Time Resolution. ACS Appl. Mater. Interfaces 13, 47629–47641 (2021).

30. Head, A. R. & Bluhm, H. Ambient Pressure X-Ray Photoelectron Spectroscopy. Encycl. Interfacial Chem. Surf. Sci. Electrochem. 13–27 (2018) doi:10.1016/B978-0-12-409547-2.10924-2.

31. Garcia-Martinez, F. et al. Catalytic Oxidation of CO on a Curved Pt(111) Surface: Simultaneous Ignition at All Facets through a Transient CO-O Complex**. Angew. Chemie Int. Ed. 59, 20037–20043 (2020).

32. Shavorskiy, A. et al. Gas Pulse-X-Ray Probe Ambient Pressure Photoelectron Spectroscopy with Submillisecond Time Resolution. ACS Appl. Mater. Interfaces 13, 47629–47641 (2021).

33. Toyoshima, R. et al. A high-pressure-induced dense CO overlayer on a Pt(111) surface: a chemical analysis using in situ near ambient pressure XPS. Phys. Chem. Chem. Phys. 16, 23564–23567 (2014).

34. Yu, Y. et al. Chemical states of surface oxygen during CO oxidation on Pt(1 1 0) surface revealed by ambient pressure XPS. J. Phys. Condens. Matter 29, 464001 (2017).

35. Miller, D. et al. Different reactivity of the various platinum oxides and chemisorbed oxygen in CO oxidation on Pt(111). J. Am. Chem. Soc. 136, 6340–6347 (2014).

36. Björneholm, O. et al. Overlayer structure from adsorbate and substrate core level binding energy shifts: CO, CCH3 and O on Pt(111). Surf. Sci. 315, L983–L989 (1994).

37. Hecq, M., Hecq, A., Delrue, J. P. & Robert, T. Sputtering deposition, XPS and X-ray diffraction characterization of oxygen-platinum compounds. J. Less Common Met. 64, P25–P37 (1979).

38. Su, H. et al. Probing the surface chemistry for reverse water gas shift reaction on Pt(1 1 1) using ambient pressure X-ray photoelectron spectroscopy. J. Catal. 391, 123–131 (2020).

39. Held, G., Jones, L. B., Seddon, E. A. & King, D. A. Effect of oxygen adsorption on the chiral Pt{531} surface. J. Phys. Chem. B 109, 6159–6163 (2005).

40. Miller, D. J. et al. Oxidation of Pt(111) under near-ambient conditions. Phys. Rev. Lett. 107, 195502 (2011).

41. Butcher, D. R. et al. In situ oxidation study of Pt(110) and its interaction with CO. J. Am. Chem. Soc. 133, 20319–20325 (2011).

42. Légaré, P. et al. On the interaction of O2 with Pt(111) and Pt(557) surfaces: core-level shift study using conventional and synchrotron radiation sources. Surf. Sci. 198, 69–78 (1988).

43. Ono, L. K., Yuan, B., Heinrich, H. & Roldan Cuenya, B. Formation and thermal stability of platinum oxides on size-selected platinum nanoparticles: Support effects. J. Phys. Chem. C 114, 22119–22133 (2010).

44. Kuribayashi, K. & Kitamura, S. Preparation of Pt-PtOx thin films as electrode for memory capacitors. Thin Solid Films 400, 160–164 (2001).

45. Shimizu, S. et al. Site-specific chemical states of adsorbed CO on Pt(997): A high resolution XPS study. Surf. Sci. 608, 220–225 (2013).

46. Tao, F. et al. Break-Up of Stepped Platinum Catalyst Surfaces by High CO Coverage. Science (80-.). 327, 850–853 (2010).
